# Nigerian malaria: the problems and the fight

**DOI:** 10.1186/1475-2875-11-S1-P122

**Published:** 2012-10-15

**Authors:** Emmanuel U Okeke

**Affiliations:** 1 King Emerson Medical Foundation, Lagos, Nigeria 23401

## 

Malaria is one of the world’s top killer diseases, especially for the young children. Malaria has remained a major threat to public health and economic development in the tropical and subtropical regions of the world. Attempts to control or completely eradicate the disease have failed massively as a result of well-known resistance to drugs for the malaria parasite and to insecticides for the vector and the situation has become life-threatening. Though there is collaborative energy both at the international, national and individual level to fight the disease by developing vaccines and new drugs, no-one has produced any permanent result yet. In the field of science, education about understanding the disease has recorded a tremendous progress.

After HIV/AIDS, malaria is the second leading cause of death in Africa. It is believe that nearly 1 in every 5 deaths among kids in Africa is as a result of malaria. In my country Nigeria, malaria is a major public health problem where it accounts for more cases and deaths than any country world over. About 97% of my country’s population is at risk for malaria because of their location. It is only 3% of Nigeria’s population live in the malaria free zones. Malaria alone accounts for more than 300,000 deaths each year in Nigeria. This estimate is well above the 215,000 deaths each year from HIV/AIDS. If the above accounts for Nigeria alone how about other 29 Sub-Saharan African countries which together accounts for 90% of the world wide malaria deaths?

The vaccine, which has been promised to be ‘just round the corner’ for many years, remains elusive. It is important to ask why this is so, when effective vaccines exist for many other infectious diseases. What are the reasons for the slow rate of progress, and what has been learned from the first clinical trials of candidate malaria vaccines? What are the remaining challenges, and what strategies can be pursued to address them? The remaining major challenge is poverty. About 70% of Nigeria population lives in poverty. Should any permanent remedy be found any time soon, these Sub-Saharan African countries like Nigeria should be the starting point in the fight to eliminating malaria!

